# A reassessment of the impact of substance use disorder on outcomes in adolescent sepsis

**DOI:** 10.1186/s13613-025-01555-8

**Published:** 2025-10-06

**Authors:** Xueneng Yang, Ruijuan Li

**Affiliations:** 1 Department of Traumatology, The Second Affiliated Hospital of Kunming Med ical University, Kunming, Yunnan China; 2 Department of Burn, The Second Affiliated Hospital of Kunming Medical Univ ersity, Kunming, Yunnan China

*Dear Editor*,

Substance Use Disorder (SUD) in adolescents continues to increase, becoming an important public health problem affecting their physical and mental health [1]. Although numerous studies have explored the impact of SUD on health outcomes in adult patients, there is a lack of systematic studies on the impact of SUD on adolescent populations, especially when they have severe infections such as sepsis. Havell [2] et al.‘s recent study published in Annals of intensive care concluded that patients with SUD have higher organ dysfunction and severe intervention needs, providing important empirical evidence for understanding the relationship between SUD and infectious severity. However, there are still issues worthy of further exploration.

First, the authors used all-cause mortality within one year as a primary outcome, but did not clarify whether the deaths were directly related to sepsis or SUD. Although all-cause mortality is objective and easy to obtain, its low specificity may lead to confounding. It can include deaths unrelated to the studied condition, such as drug overdose, suicide, trauma, or progression of non-infectious chronic diseases.

Second, the study did not adjust for the source or type of infection when building the multivariable models, which is a key confounding factor. Different infection sites—such as the lungs, urinary tract, or abdomen—can significantly affect the course of sepsis, patterns of organ dysfunction, and the need for intensive care [3]. Without accounting for these differences, the observed associations may reflect the effects of infection type rather than the impact of SUD, leading to confounding bias.

Finally, the study treated SUD as a binary variable based only on the presence of ICD-10 diagnostic codes, without considering severity, frequency of use, type of substance, or withdrawal status. However, SUD is clinically heterogeneous, and its effects on immune function, metabolism, and infection risk can vary greatly between individuals [4]. Simplifying SUD as a “yes/no” condition may overlook potential dose-response relationships.

In summary, the study by Havell [2] et al. addresses the association between adolescent sepsis and substance use disorder (SUD), filling an important gap in current research. Future studies, where data permit, should further classify causes of death to distinguish between infection-related and non-infection-related mortality. Infection source or type should also be included as a covariate to account for its influence on disease course and outcomes. Additionally, refining the definition of SUD exposure by incorporating substance type, frequency, and severity may allow for a more accurate assessment of its true impact through a more nuanced, multi-level variable.

## Data Availability

All data is available in the manuscript.

## Declarations

### Patient and public involvement

No patient involved.
